# Use of Transcriptomics to Reveal the Joint Immunotoxicity Mechanism Initiated by Difenoconazole and Chlorothalonil in the Human Jurkat T-Cell Line

**DOI:** 10.3390/foods13010034

**Published:** 2023-12-21

**Authors:** Yun-Cheng Li, Shu-Yan Liu, Hou-Ru Li, Fan-Bing Meng, Jing Qiu, Yong-Zhong Qian, Yan-Yang Xu

**Affiliations:** 1 Institute of Quality Standards and Testing Technology for Agro-Products, Chinese Academy of Agricultural Sciences, Beijing 100081, China; liyunchengs@126.com (Y.-C.L.); qiujing@caas.cn (J.Q.); xuyanyang@caas.cn (Y.-Y.X.); 2 College of Food and Biological Engineering, Chengdu University, Chengdu 610106, China; lsy999579@163.com (S.-Y.L.); masterhouru@163.com (H.-R.L.); mfb1020@163.com (F.-B.M.)

**Keywords:** chlorothalonil, difenoconazole, joint immunotoxicity, Jurkat T cells line, transcriptomics

## Abstract

It is very important to evaluate the immunotoxicity and molecular mechanisms of pesticides. In this study, difenoconazole and chlorothalonil were evaluated for immunotoxicity by using the human Jurkat T-cell line, and the EC_50_ were 24.66 and 1.17 mg/L, respectively. The joint exposure of difenoconazole and chlorothalonil showed a synergistic effect at low concentrations (lower than 10.58 mg/L) but an antagonistic effect at high concentrations (higher than 10.58 mg/L). With joint exposure at a concentration of EC_10_, the proportion of late apoptotic cells was 2.26- and 2.91-fold higher than that with exposure to difenoconazole or chlorothalonil alone, respectively. A transcriptomics analysis indicated that the DEGs for single exposure are associated with immunodeficiency disease. Single exposure to chlorothalonil was mainly involved in cation transportation, extracellular matrix organization, and leukocyte cell adhesion. Single exposure to difenoconazole was mainly involved in nervous system development, muscle contraction, and immune system processes. However, when the joint exposure dose was EC_10_, the DEGs were mainly involved in the formation of cell structures, but the DEGs were mainly involved in cellular processes and metabolism when the joint exposure dose was EC_25_. The results indicated that the immunotoxicological mechanisms underlying joint exposure to difenoconazole and chlorothalonil are different under low and high doses.

## 1. Introduction

The use of pesticides is the most direct, economic, and effective measure to prevent and control pests, fungi, and rodents in modern agricultural production. They can also serve as plant regulators for promoting plant growth or inhibiting weed growth [1]. Without pesticides, the food supply would be insufficient to meet the growth of the world population and the loss of farmland [2]. However, the long-term and continuous spraying of pesticides will make pests resistant; thus, many new pesticides are developed and applied to crops [2], which causes an increasing number of pesticide residues and their degradations to enter the environment and food matrices, accumulate in human tissue, and pose a potential threat to human health [3]. Therefore, it is vital to carry out pesticide toxicity studies and fully understand the toxicity mechanism and endpoint of pesticides because it plays an important role in safety assessment and guiding the application of pesticides.

Currently, pesticide toxicity studies mainly focus on teratogenic effects, oxidative stress, endocrine disruption, neurotoxicity, reproductive toxicity, and cardiotoxicity [4,5,6]. Although in the mid-1970s, the relevant departments of many countries tried to formulate evaluation strategies for immunotoxicity [7], research on the immunotoxicity of pesticides is still insufficient. Substances with immunotoxicity will cause adverse effects to the body, such as decreased immunocompetence, inappropriate immunostimulation, tissue inflammation, and organ damage [8]. The first two effects are specific immunity, and the third is nonspecific immunity. The immune system is the first barrier of the human body to foreign hazards. Generally, a low-dose exposure to pesticides may not induce cytotoxicity but interferes with many kinds of general or immune-specific signaling pathways and alters cell function [8,9,10]. These effects are potential and not obvious, but the dysregulation of the immune system by pesticides may be highly associated with a predisposition to different types of disease because the immune system is responsible for the defense against disease [11]. Therefore, it is very important to evaluate the immunotoxicity and molecular mechanisms of pesticides.

Currently, pesticide risk assessments are mostly based on toxicity evaluations of single chemical agents [12]. However, due to the spraying of multiple pesticides and the accumulation of different food chains, human populations are usually exposed to a combination of two or more different pesticides simultaneously through their foods [13,14]. When humans are exposed to multiple pesticides at the same time, joint toxicity may occur. The joint toxic effects are mainly caused by the physicochemical properties, physiology characteristics, toxic endpoint, and ecological behaviors of the pesticides, including independent effects, potentiating effects, synergistic effects, and antagonistic effects [15]. Even more complicated, these effects coexist in different concentration ranges of the same pesticide combinations [16]. Therefore, studying the possible joint toxicity of different pesticides is of great significance for accurately understanding the risks of pesticide mixtures [12].

Classical toxicological studies are performed by using animal experiments [17]. This approach is costly, time-consuming, and under constant review [18]. Therefore, there is a strong need for a rapid mechanism-based strategy for risk assessment by using cell lines [19]. In this context, toxicogenomics (such as transcriptomics, epigenomics, or metabolomics, etc.) has made a promising contribution to risk assessment in the past two decades [18]. Using the toxicogenomics method, human cell lines are usually chosen as an up-to-date in vitro model of human organs, in connection with phenotypic evaluation and transcriptomics analysis, to unravel potential toxic properties. This strategy could be used to decide whether to terminate or continue animal experiments, matching the need to apply the ‘3Rs’ concept (replacement, reduction, and refinement) [19]. Among the cell lines, the human Jurkat T-cell line is frequently used in immunotoxicity evaluation because of its well-established reliability. Previously, Lee et al. [20] assessed the immunotoxicity of fludioxonil by using the human Jurkat T-cell line, and found that fludioxonil could induce immunotoxicity through apoptosis and cell cycle arrest. Therefore, in the present study, an in vitro model system human Jurkat T-cell line was chosen to assess the immunotoxicity of typical pesticides in leaf vegetables. Two pesticides, difenoconazole and chlorothalonil, were found to have immunotoxicity. Therefore, single and joint immunotoxicity and their mechanisms were evaluated by using the transcriptomics method. To our knowledge, this is the first study to use a human immune cell line to evaluate the joint immunotoxicity of pesticides based on a transcriptomics method.

## 2. Materials and Methods

### 2.1. Chemicals and Reagents

All pesticides shown in Table S1 were obtained from Alta Scientific Ltd. (Tianjin, China). A 11,000 mg/L stock solution of the pesticides was prepared in acetone (Merck & Co., Darmstadt, Germany) and maintained at −80 °C. RPMI-1640 medium, penicillin/streptomycin, phosphate-buffered saline (PBS), and fetal bovine serum (FBS) were all obtained from HyClone (Logan, UT, USA). The Cell Counting Kit-8 was obtained from Dojindo (Kumamoto, Japan). The Annexin V-FITC/PI detection kit was obtained from Abbkine (Wuhan, China). The Mycoplasma Stain Kit was obtained from Sigma Aldrich (Saint Louis, MO, USA), the TruSeqTM RNA sample preparation Kit was obtained from Illumina (San Diego, CA, USA), and the PrimeScript RT Reagent Kit was obtained from Beyotime Biotechnology (Shanghai, China). Unless otherwise stated, the other reagents used in this study were of the highest purity available. 

### 2.2. Jurkat Cell Culture

The human T-lymphocyte cell line (Jurkat T cells) was purchased from the American Type Culture Collection (ATCC TIB-152, Manassas, VA, USA). After resuscitation, 2 × 10^4^ cells were inoculated in a 25 cm^2^ cell culture flask containing 10 mL RPMI-1640 medium, 10% (*v*/*v*) heat-inactivated FBS, 100 U/mL penicillin sodium, and 100 μg/mL streptomycin solution. The cells were cultured in a cell culture incubator containing 5% CO_2_ at 37 °C, maintained in the exponential growth phase by subculture 2–3 d intervals, and then used for subsequent experiments. The absence of mycoplasma was checked routinely using the Mycoplasma Stain Kit [21].

### 2.3. Immunotoxicity Evaluation of Single Pesticide Exposure

The immunotoxicity of single pesticide exposure was evaluated through cell viability assessment using the Cell Counting Kit-8 (CCK-8) (Dojindo, Kumamoto, Japan) according to the manufacturer’s instructions. Briefly, 100 μL of activated Jurkat T cells was seeded into a 96-well plate (2 × 10^4^ cells/well) and exposed for 36 h to a single pesticide at final concentrations of 0.5, 5, 25, 50, 100, 250, and 500 mg/L. The final dissolvent (acetone) concentration was adjusted to the same concentration and less than 0.1%, which exerted no effect on cell viability [21]. The cells exposed to 0.1% sterile ultrapure water and acetone were used as the blank and control groups, respectively. After exposure, a 10% (*v*/*v*) CCK-8 solution was added to the well and re-incubated for 2 h [21]. Absorbance was measured at 450 nm in a ReadMax 500F enzyme-labeled instrument (Shanpu Biotechnology Co., Ltd., Shanghai, China). Cell viability was calculated as Equation (1).

(1) $$Cell\;viability=\frac{A_{1}-A_{2}}{A_{3}-A_{2}}$$

where A_1_ is the absorbance of the test group, A_2_ is the absorbance of the blank group, and A_3_ is the absorbance of the control group. Concentration–response curves were plotted, and the 50%, 25%, and 10% effective concentration (EC_50_, EC_25_, and EC_10_, respectively) values were then calculated using a sigmoidal dose–response curve equation [22].

### 2.4. Joint Immunotoxicity Evaluation

The combined effects of difenoconazole and chlorothalonil were predicted according to the description of Chou et al. [23] by using a combination index (CI) model derived from the median effect principle. The cells were exposed to single and mixed pesticides with constant ratio combinations (0.5×, 0.75×, 1×, 1.5×, and 2 × EC_50_), and the cell viability was measured. The cell viabilities were introduced into CompuSyn software (version 1.0) to calculate the CI. CI < 1, =1, and >1 indicate synergism, additive effect, and antagonism, respectively.

### 2.5. Cell Apoptosis Analysis

Cell apoptosis was evaluated according to the description of Verschoor et al. [24] by using an Annexin V-FITC/PI detection kit. Four milliliters of Jurkat T cells were seeded into a 6-well plate (2 × 10^5^ cells/mL) and exposed for 36 h to single (difenoconazole or chlorothalonil) or mixed pesticides (difenoconazole and chlorothalonil) at final concentrations of EC_50_, EC_25_, and EC_10_. The cells were collected and washed using PBS buffer to remove the medium, and then they were resuspended in binding buffer and incubated with Annexin V-FITC solution and PI solution at room temperature for 15 min. Apoptotic cells were detected using a MoFlo Astrios^EQ^ flow cytometer (Beckman Coulter, Inc., Brea, CA, USA).

### 2.6. RNA Extraction and Sequencing

The activated Jurkat T cells were inoculated in a 75 cm^2^ cell culture flask with 60 mL medium at an initial concentration of 5 × 10^6^ cells/bottle and exposed for 36 h to single or mixed pesticides at final concentrations of EC_10_ and EC_25_, respectively. Upon removal of the culture medium after exposure, the cells were disrupted and homogenized in TRIzol^®^ reagent (Thermo Fisher, Waltham, MA, USA), and then RNA was isolated according to the operating manual [21]. Genomic DNA was eliminated using DNase I (TaKaRa, Dalian, China). Then, the RNA quality was evaluated using an RNA 6000 Nano LabChip Kit and a 2100 Bioanalyzer (Agilent Technologies, Santa Clara, CA, USA). The RNA quantity was determined using the NanoDrop ND-2000 (Waltham, MA, USA). Only RNA samples meeting the quality requirements for library construction (OD260/280 = 1.8~2.2, OD260/230 ≥ 2.0, RIN ≥ 6.5, 28S:18S ≥ 1.0, >1 μg) were applied to construct the sequencing library.

The transcription library was prepared according to the operating manual of the TruSeqTM RNA sample preparation kit. The libraries were size-selected for cDNA target fragments of 300 bp on 2% Low Range Ultra Agarose followed by PCR amplification using Phusion DNA polymerase (NEB, Waltham, MA, USA) for 15 PCR cycles [21]. After quantification using TBS-380 (Turner BioSystems, Inc. Sunnyvale, CA, USA), a paired-end RNA sequencing library was obtained by using a Nova Seq 6000 sequencer (2 × 150 bp read length).

### 2.7. Read Mapping and Differentially Expressed Gene Analysis

The raw paired-end reads were trimmed and quality controlled by SeqPrep (https://github.com/jstjohn/SeqPrep, accessed on 25 January 2022) and Sickle (https://github.com/najoshi/sickle, accessed on 25 January 2022) with default parameters. The clean reads of each sample were sequenced and aligned with the specified reference genome (Homo_sapiens, http://asia.ensembl.org/Homo_sapiens/Info/Index, accessed on 25 January 2022). The mapped reads of the sample were assembled using StringTie (https://ccb.jhu.edu/software/stringtie/index.shtmlt=example, accessed on 25 January 2022) in a reference-based approach [25].

To identify differentially expressed genes (DEGs), the expression level of each transcript was normalized according to the fragments per kilobases per million reads (FPKM). RSEM (http://deweylab.biostat.wisc.edu/rsem/, accessed on 13 March 2022) [26] was applied to quantify gene abundances. Essentially, differential expression analysis was performed using DESeq2 [27], with |log2FC| ≥ 1 and *p* value ≤ 0.05 regarded to be DEGs. Functional enrichment analysis, including gene ontology (GO, http://www.geneontology.org, accessed on 15 March 2022) and Kyoto Encyclopedia of Genes and Genomes (KEGG, http://www.genome.jp/kegg/, accessed on 15 March 2022) analysis, was performed to identify which DEGs were significantly enriched in GO terms and metabolic pathways [28].

### 2.8. Quantitative Real-Time PCR (qRT–PCR)

Total RNA from Jurkat T cells without exposure and with single (difenoconazole or chlorothalonil) or joint pesticide (difenoconazole and chlorothalonil) exposure for 36 h was used for qRT–PCR analysis. The glyceraldehyde-3-phosphate dehydrogenase encoded gene *GAPDH* was used as the reference gene, and the expression values of the genes were normalized on the relative expression of *GAPDH*. The primers were designed with Sangon Biotech (https://www.sangon.com/, (accessed on 9 August 2023) and are presented in Supplementary Table S2. The total RNA was reverse-transcribed according to the instruction of the PrimeScript RT Reagent Kit (Takara, Osaka, Japan) with gDNA Eraser. The reactions were prepared on a StepOne Plus^TM^ Real-time PCR detection system (ABI, Norwalk, CT, USA), with a total volume of 10 μL reaction: 3 μL of 1:2 diluted template, 1 μL of each primer (5 μM), and 5 μL of 2× Fast SYBR^®^ Green Master Mix (ABI). Baseline, threshold cycles (Ct), and statistical analysis were automatically determined using the StepOne Plus^TM^ Software version 2.3 (ABI), and the 2^−ΔΔCT^ method was applied to analyze the relative gene expression levels [3].

### 2.9. Statistical Analysis

The transcriptomics analysis was performed in six biologically independent experiments (*n* = 6), and the other analyses were conducted in three independent experiments (*n* = 3). The GO enrichment analysis was performed using goatools software (version 0.6.5). R statistical package software (version 1.6.2) was applied for unsupervised principal component analysis (PCA) and hierarchical clustering analysis. Data were recorded as the mean ± SD. All statistical analyses were performed using SPSS version 18.0 software (IBM). The values were compared with one-way ANOVA followed by Duncan’s test. *p* < 0.05 was considered statistically significant.

## 3. Results and Discussion

### 3.1. Immunotoxicity Screening of Pesticides Based on Human Jurkat Cells

**Figure 1:**
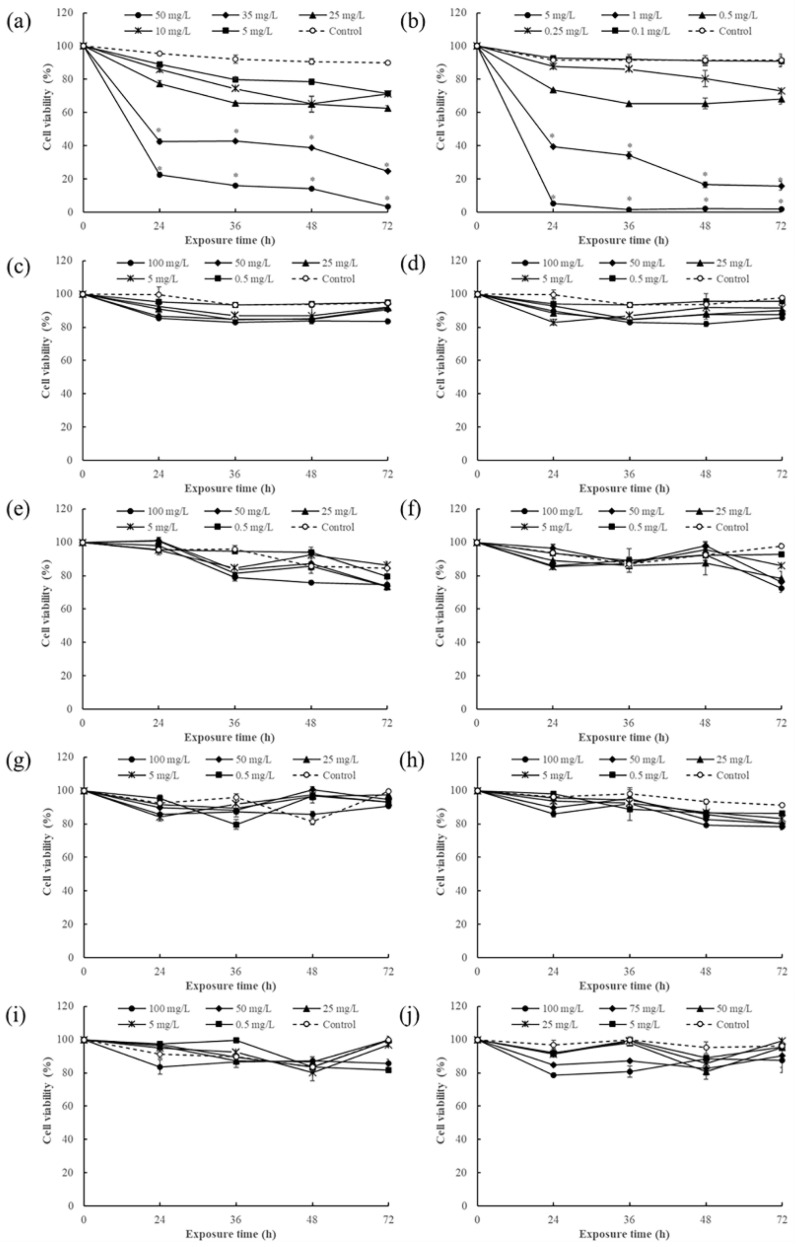
Immunotoxicity screening of the pesticides based on the Jurkat T-cell line. (**a**) Difenoconazole, (**b**) chlorothalonil, (**c**) bifenthrin, (**d**) cypermethrin, (**e**) dimethoate, (**f**) omethoate, (**g**) imidacloprid, (**h**) acetamiprid, (**i**) iprodione, and (**j**) chlorpyrifos.

Toxicological evaluation has important guiding significance for the scientific application of pesticides during agricultural production and the protection of human health [3]. The immune system is the first line of defense against foreign hazardous chemicals within the human body [21], so immunotoxicity evaluations of pesticide residues are very important for a comprehensive pesticide residue risk assessment. In the present study, the immunotoxicity of nine commonly used pesticides in vegetable farming (difenoconazole, chlorothalonil, bifenthrin, cypermethrin, dimethoate, omethoate, imidacloprid, acetamiprid, and iprodione) and one prohibited but often detected pesticide (chlorpyrifos) were investigated by using human Jurkat cells. As shown in Figure 1, eight pesticides (bifenthrin, cypermethrin, dimethoate, omethoate, imidacloprid, acetamiprid, iprodione, and chlorpyrifos) did not show significant cytotoxicity under exposure concentration and time. However, two pesticides (difenoconazole and chlorothalonil) showed obvious inhibitory effects on Jurkat cell growth in a dose- and time-dependent manner.

### 3.2. Effect of Single Pesticide Exposure on Cell Viability

**Figure 2:**
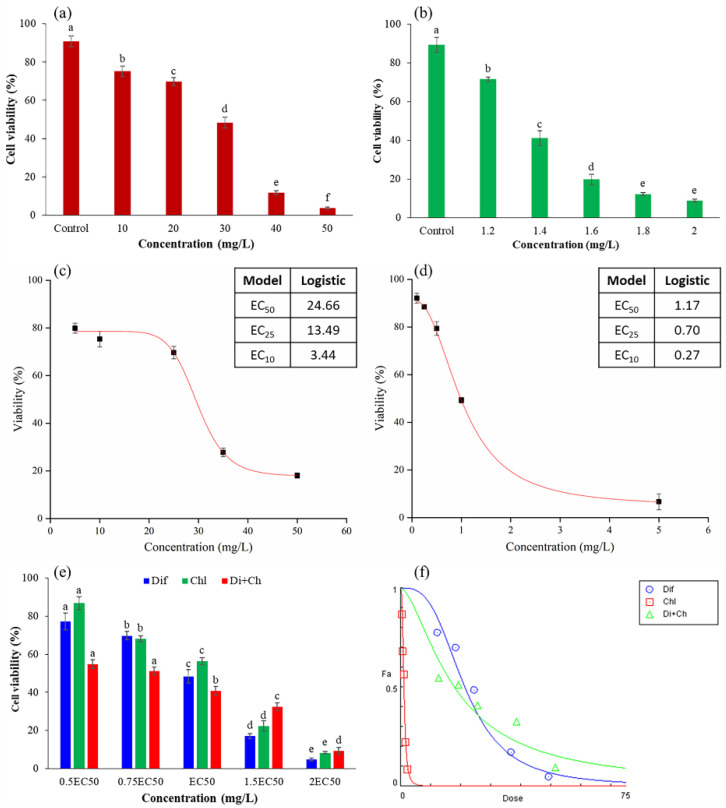
Effect of single and joint exposure to pesticides on Jurkat T-cell viability. (**a**,**b**) Jurkat cells exposed for 36 h to difenoconazole and chlorothalonil, respectively; (**c**,**d**) nonlinear curve fitting results of different effective concentrations (ECs) using the results of (**a**,**b**), respectively; (**e**) effects of joint exposure to difenoconazole and chlorothalonil on Jurkat T-cell viability; (**f**) combination index (CI) plot for the joint exposure to difenoconazole and chlorothalonil in Jurkat T cells. Dif: difenoconazole, Ch: chlorothalonil, Di+Ch: joint exposure to difenoconazole and chlorothalonil. Values with different superscripted lowercase letters of the same color bars in (**a**,**b**,**e**) are significantly different (*p* < 0.05).

As shown in Figure 2a,b, cell activity gradually decreased with increasing concentrations of difenoconazole and chlorothalonil. The 50% effective concentration (EC_50_) values according to the sigmoidal dose–response curve equation for difenoconazole and chlorothalonil were 24.66 and 1.17 mg/L, respectively (Figure 2c,d), and further verification tests were also consistent with the model calculation results. The cell viabilities for difenoconazole and chlorothalonil under EC_50_ concentrations were 48.4% and 56.3%, respectively (Figure 2c). A previous study indicated that difenoconazole and chlorothalonil are carcinogens for humans, and they can induce embryonic and developmental toxicity, as well as estrogenic endocrine-disrupting effects for aquatic animals [29]. Liu et al. [30] and Guerreiro et al. [31] pointed out that difenoconazole and chlorothalonil exposure could induce immunotoxicity in carp and marine bivalves. However, few studies have reported that difenoconazole has potential immunotoxicity in humans. According to our results, these two pesticides have potential immunotoxicity to the human body.

### 3.3. Effects of Combined Exposure to Difenoconazole and Chlorothalonil on Cell Viability

Many studies have pointed out that when humans are simultaneously exposed to some pesticides, the toxicity of pesticides may be enhanced or weakened [15]. Therefore, the combined toxicities of pesticides have gradually received attention in recent years. As shown in Figure 2f, the CI values ranged from 0.47 to 0.96 at low total dose exposures (2.06~9.05 mg/L), which indicated that the combined effect was synergistic at low concentrations. When the combination concentration was increased to 10.58 mg/L, the CI values were higher than 1, indicating that an antagonistic effect existed at high total dose exposures. Different pesticide combinations have different combined toxicities at different concentrations, which has been reported in previous studies [6]. It is generally considered that the combined effects of pesticides may be related to the interactions between pesticides or the target and modes of action [15]. However, why different concentrations of the same pesticide combinations exhibit different combined toxicities needs further exploration.

### 3.4. Effects of Pesticide Exposure on Cell Apoptosis

**Figure 3:**
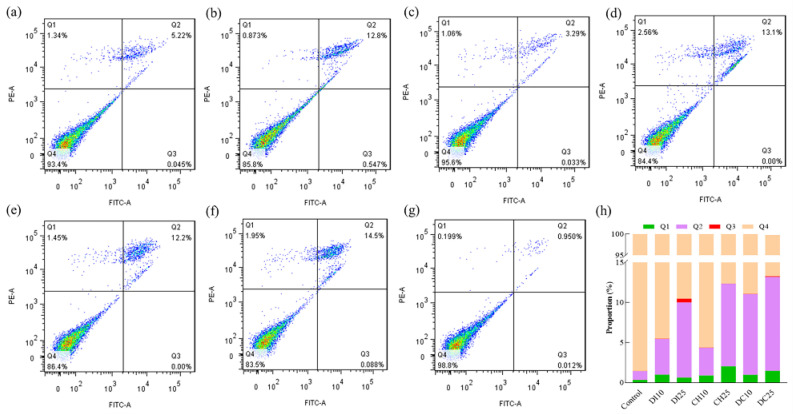
Single and joint exposure to difenoconazole and chlorothalonil induced apoptosis in Jurkat T cells. (**a**) EC_10_ of difenoconazole, (**b**) EC_25_ of difenoconazole, (**c**) EC_10_ of chlorothalonil, (**d**) EC_25_ of chlorothalonil, (**e**) joint exposure dose of EC_10_, (**f**) joint exposure dose of EC_25_, (**g**) control, and (**h**) statistics of the apoptosis data of two independent experiments. Q1, Q2, Q3, and Q4 of the flow cytometry graph indicate dead cells, late apoptotic cells, early apoptotic cells, and normal cells, respectively. CH10 and CH25 indicate that the chlorothalonil exposure doses were EC_10_ and EC_25_, respectively; DI10 and DI25 indicate that the difenoconazole exposure doses were EC_10_ and EC_25_, respectively; and DC10 and DC25 indicate a joint exposure to difenoconazole and chlorothalonil at doses of EC_10_ and EC_25_, respectively.

As shown in Figure 3, pesticide exposure resulted in a significant increase in cell death and apoptosis compared with the control group. At low exposure concentrations (the EC_10_ dose), the proportion of late apoptotic cells in the difenoconazole group was slightly higher than that in the chlorothalonil group. However, when the cells were jointly exposed to the two pesticides, the proportion of late apoptotic cells was 2.26- and 2.91-fold higher than that after exposure to difenoconazole or chlorothalonil alone, respectively (Figure 3h). This result indicated that the combined exposure to difenoconazole and chlorothalonil has a synergistic effect on Jurkat T cells, which is consistent with the cell viability assessment results. However, under high exposure concentrations (dose of EC_25_), the proportion of cell apoptosis significantly increased compared to that of the control and low exposure concentrations. Although the apoptosis rate of the combined exposure group (average of 11.69%) was higher than that of the single exposure group (average of 10.30% and 9.38% for difenoconazole and chlorothalonil, respectively), this difference was not significant (Figure 3h), indicating that the synergistic effect was weakened or even antagonistic, which is also consistent with the cell viability assessment results.

### 3.5. Transcriptomics Analysis

#### 3.5.1. Overview

The immune system can affect the function of various organs in the human body, so the potential immunotoxicity caused by pesticides should be taken seriously [32]. Presently, there are few studies on the immunotoxicity mechanism of pesticides [21]. Therefore, in the present study, transcriptomics was used to examine the effects of single and joint exposure to difenoconazole and chlorothalonil on the gene expression of Jurkat T cells. As shown in Tables S3 and S4, the OD260/280 and OD260/230 were in the range of 1.91~1.99 and 2.23~2.30, respectively, which indicates good RNA quality. During RNA sequencing, the error rate was less than 0.03%, and the Q30 base was higher than 93%, which suggests that the quality of the sequencing was high enough for further analysis [21].

**Figure 4:**
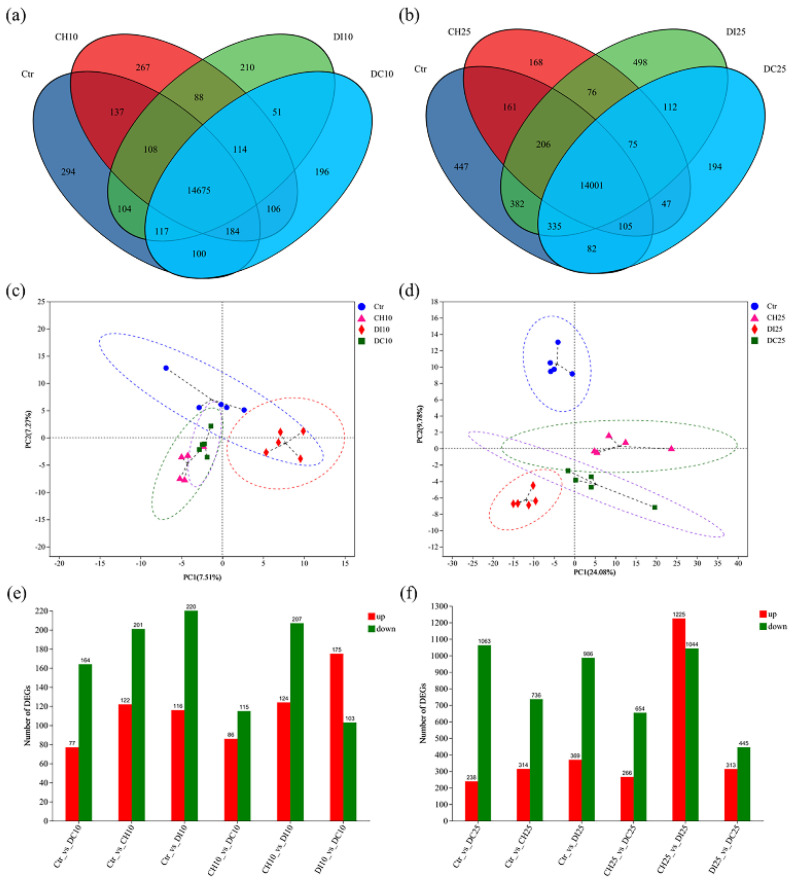
Statistical overview of the transcriptomics data. (**a**,**b**) Venn diagram of the significantly differentially expressed genes; (**c**,**d**) principal component analysis (PCA) of the significantly differentially expressed genes; (**e**,**f**) statistics of the significantly differentially expressed genes for each comparison. CH10 and CH25 indicate that the chlorothalonil exposure doses were EC_10_ and EC_25_, respectively; DI10 and DI25 indicate that the difenoconazole exposure doses were EC_10_ and EC_25_, respectively; and DC10 and DC25 indicate a joint exposure to difenoconazole and chlorothalonil at doses of EC_10_ and EC_25_, respectively; Ctr: Control.

In total, 16,751 and 16,889 genes were identified under exposure concentrations of EC_10_ and EC_25_ (Figure 4a,b), respectively. The PCA results indicated that the identified genes can distinguish samples from different groups (Figure 4c,d). Under an exposure dose of EC_10_, the chlorothalonil group and joint exposure group are close together, which may be because in the joint exposure group, the toxicity of chlorothalonil dominates. Under an exposure dose of EC_25_, different samples were divided into characteristic groups by PCA, which indicated that the transcriptomics were different between the groups [33]. To further reveal the immunotoxicity of the pesticides, DEGs (|log2FC| ≥ 1 and *p* value ≤ 0.05) were identified. As shown in Figure 4e,f and Tables S5–S14, under an exposure dose of EC_10_, 323, 336, 241, 331, 201, and 278 DEGs were identified in the comparison of Ctr_vs_CH10, Ctr_vs_DI10, Ctr_vs_DC10, CH10_vs_DI10, CH10_vs_DC10, and DI10_vs_DC10, respectively. Under an exposure dose of EC_25_, the DEGs increased to 1050, 1355, 1301, 2269, 942, and 758 for the comparison of Ctr_vs_CH25, Ctr_vs_DI25, Ctr _vs_DC25, CH25_vs_DC25, and DI25_vs_DC25, respectively.

#### 3.5.2. GO Enrichment Analysis of DEGs

**Figure 5:**
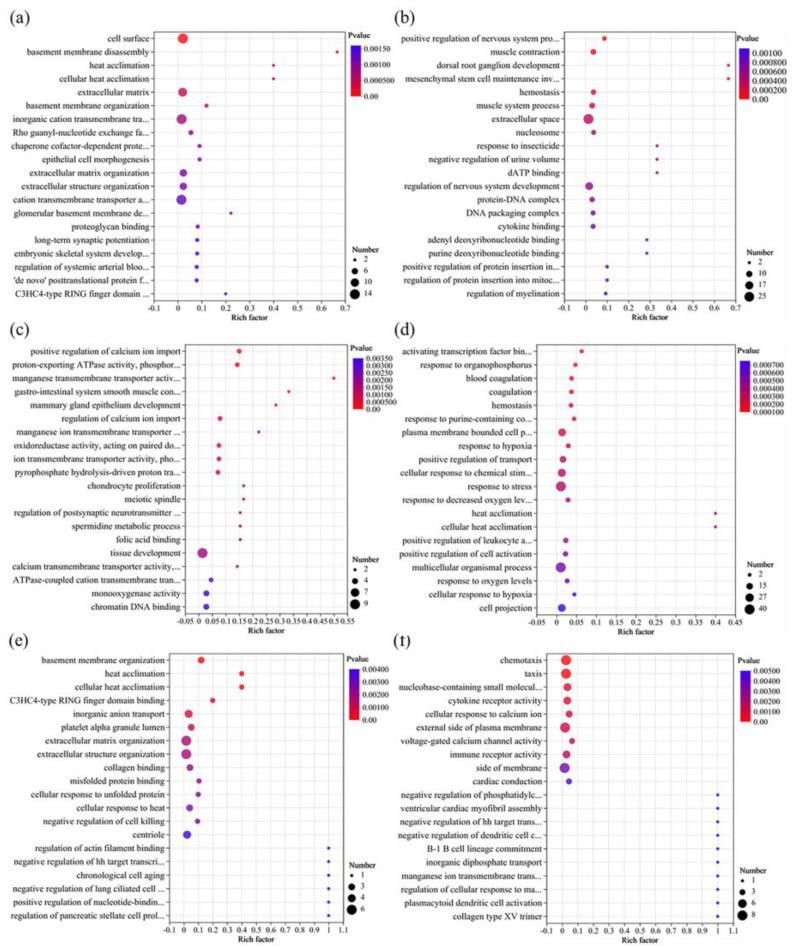
Gene ontology enrichment analysis of the significantly differentially expressed genes at single and joint exposure doses of EC_10_. (**a**) Ctr_vs_CH10, (**b**) Ctr_vs_DI10, (**c**) Ctr_vs_DC10, (**d**) CH10_vs_DI10, (**e**) CH10_vs_DC10, and (**f**) DI10_vs_DC10. CH10 and CH25 indicate that the chlorothalonil exposure doses were EC_10_ and EC_25_, respectively; DI10 and DI25 indicate that the difenoconazole exposure doses were EC_10_ and EC_25_, respectively; and DC10 and DC25 indicate a joint exposure to difenoconazole and chlorothalonil at doses of EC_10_ and EC_25_, respectively; Ctr: Control.

**Figure 6:**
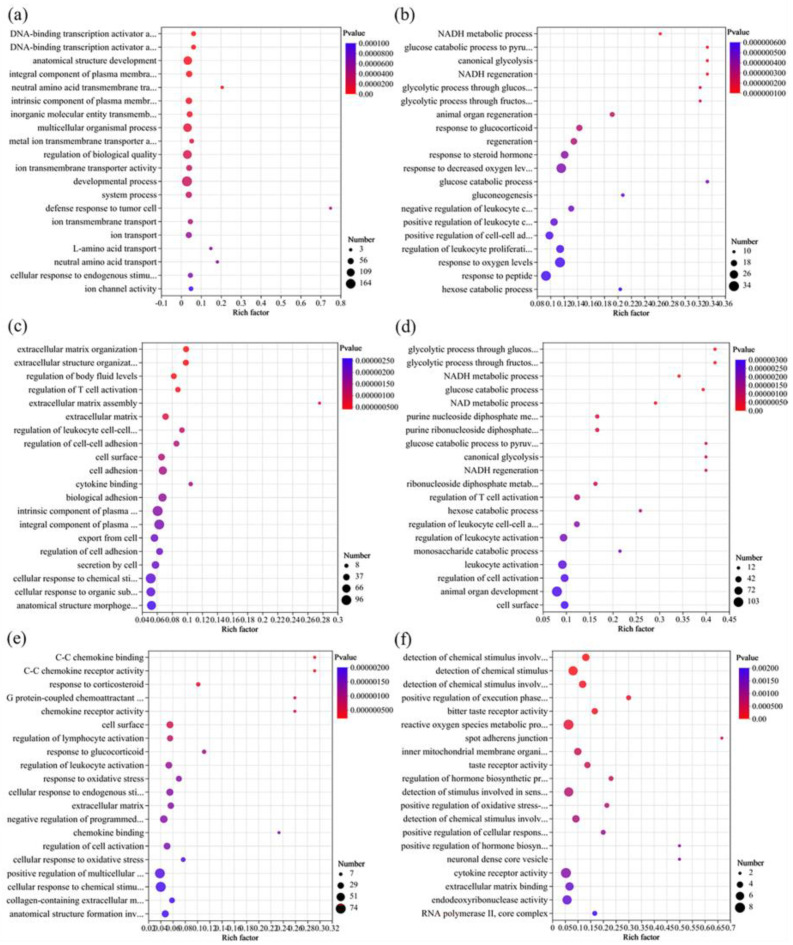
Gene ontology enrichment analysis of the significantly differentially expressed genes at single and joint exposure doses of EC_25_. (**a**) Ctr_vs_CH25, (**b**) Ctr_vs_DI25, (**c**) Ctr_vs_DC25, (**d**) CH25_vs_DI25, (**e**) CH25_vs_DC25, and (**f**) DI25_vs_DC25.

As shown in Figure 5 and Table S15, exposure to chlorothalonil mainly caused changes in cell membrane function, especially cation transport, and further affected cell growth and development. Under an exposure dose of EC_10_, the most significant top five GO terms were cellular response to chemical stimulus (9 DEGs downregulated, 11 DEGs upregulated), homeostatic process (11 DEGs downregulated, 8 DEGs upregulated), cellular response to organic substance (8 DEGs downregulated, 9 DEGs upregulated), transmembrane transporter activity (12 DEGs downregulated, 5 DEGs upregulated), and inorganic molecular entity transmembrane transporter activity (12 DEGs downregulated, 3 DEGs upregulated) (Tables S5 and S15). A previous study also suggested that chlorothalonil exposure could reduce the secretion of colonic epithelial mucus and change the gene transcription of ion transportation [34]. When the exposure dose increased to EC_25_, the GO term enrichment was similar to that of EC_10_ (Figure 6a and Table S16), and many genes were enriched in the GO terms of membrane structure and ion transport, such as inorganic molecular entity transmembrane transporter activity, ion transmembrane transporter activity, and ion transmembrane transport. In addition, cell development, such as developmental process, anatomical structure development, and system process, was also significantly affected (Figure 6a), which indicates that an increase in pesticide concentration may cause more gene expression disorders. Notably, eight genes were enriched in the negative regulation of leukocyte cell–cell adhesion (Table S16), three downregulated (*FGL2*, *GNRH1*, and *IL2RA*), and five upregulated (*ASS1*, *CD86*, *FOXJ1*, *KLF4*, and *SMAD7*). In addition, three downregulated genes (*IL2RA*, *ICOSLG*, and *CD24*) were enriched in the positive regulation of activated T-cell proliferation (Table S16). Among the DEGs, two key immune regulation genes, cluster of differentiation 24 (*CD24*, downregulated) and chymotryptic serine proteinase (*CMA1*, upregulated), were significantly differentially expressed with the same trends in the comparison of Ctr_vs_CH10 and Ctr_vs_CH25. In the human body, *CD24* expression could affect cell adhesion, which is closely related to cancer cell migration, invasion, and proliferation [35]. The downregulation may be an important reason that most of the genes (9/13) in the GO term of cell adhesion were downregulated (Table S15). *CMA1* is a good immune-related prognostic marker for gastric cancer, and it was upregulated in the high-risk prognosis of gastric cancer [36]. However, *CD24* was also downregulated after exposure to difenoconazole, so *CMA1* was considered a potential target gene for chlorothalonil exposure.

When the exposure dose was EC_10_, difenoconazole exposure mainly affected the regulation of cell and nervous system development. A previous study indicated that the exposure of zebrafish to difenoconazole could initiate neurotoxicity [37]. Moreover, muscle contraction (seven downregulated DEGs, one upregulated DEG) and muscle system processes (seven downregulated DEGs, one upregulated DEG) were also significantly affected (Tables S7 and S17). Muscle contraction is closely related to energy metabolism [38]. Therefore, when the exposure dose increased to EC_25_, the oxygen levels (response to decreased oxygen levels), nicotinamide adenine dinucleotide (NADH) metabolism, and glucose metabolism were affected (Figure 6). In addition, the DEGs were also mainly enriched in membrane construction, cellular metabolism, and signal transduction (Table S18) at an exposure dose of EC_25_. Notably, the exposure dose of EC_10_ significantly affected the immune system, especially the regulation of immune system processes (15 DEGs downregulated, 5 DEGs upregulated) and the positive regulation of immune system processes (11 DEGs downregulated, 4 DEGs upregulated) (Tables S7 and S17), but the exposure dose of EC_25_ significantly affected the immune system (such as the GO of immune system processes and the regulation of immune system processes) and leukocyte activation (such as the GO of leukocyte activation and the regulation of leukocyte activation). Among the immune-related DEGs, six genes (*TRIM15*, *TNFRSF1B*, *STAP1*, *SERPINE1*, *LGALS1*, and *HMGB1P37*) maintained the same regulatory trends at the exposure doses of EC_10_ and EC_25_. However, these genes were not significantly differentially expressed under chlorothalonil exposure, so they are potential target genes for difenoconazole exposure.

When the Jurkat T cells were jointly exposed to chlorothalonil and difenoconazole, gene expression changed. The DEGs between the joint exposure group and chlorothalonil exposure group at the dose of EC_10_ (CH10_vs_DC10) were mainly enriched in the formation of cell structures, such as membrane-enclosed lumen, intracellular organelle lumen, and extracellular structure organization (Figure 5e and Table S21). When the exposure dose increased to EC_25_, the DEGs were mainly enriched in cellular processes and cellular metabolism (Figure 6e and Table S22). However, compared to difenoconazole, the DEGs of the joint exposure group and difenoconazole exposure group at the dose of EC_10_ (DI10_vs_DC10) were different. The DEGs were mainly enriched in GO terms of cell metabolism, particularly in amino acid metabolism (Figure 5f and Table S23). When the exposure dose increased to EC_25_, the DEGs of the joint exposure group and difenoconazole exposure group (DI10_vs_DC10) were mainly related to cell development (such as cell differentiation, mitochondrial inner membrane, and DNA recombination) and cell oxidative stress (such as response to oxidative stress and reactive oxygen species metabolic process) (Figure 6f and Table S24). The above results indicated that the effects of combined exposure are different under low (EC_10_) and high (EC_25_) exposure doses, which is consistent with the cytotoxicity results of Section 3.3.

#### 3.5.3. KEGG Enrichment Analysis of DEGs

**Figure 7:**
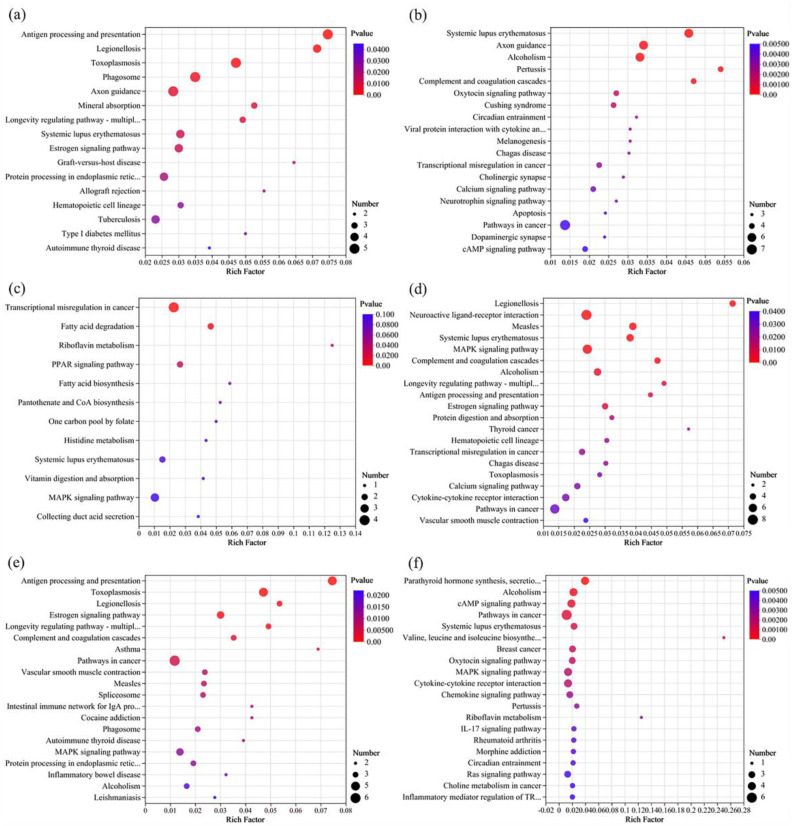
KEGG enrichment analysis of the significantly differentially expressed genes at single and joint exposure doses of EC_10_. (**a**) Ctr_vs_CH10, (**b**) Ctr_vs_DI10, (**c**) Ctr_vs_DC10, (**d**) CH10_vs_DI10, (**e**) CH10_vs_DC10, and (**f**) DI10_vs_DC10.

**Figure 8:**
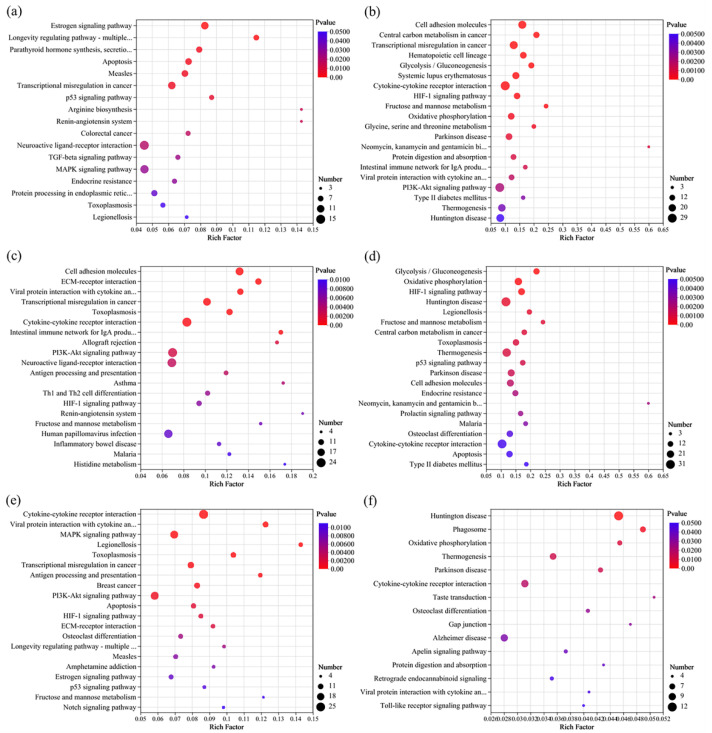
KEGG enrichment analysis of the significantly differentially expressed genes at single and joint exposure doses of EC_25_. (**a**) Ctr_vs_CH25, (**b**) Ctr_vs_DI25, (**c**) Ctr_vs_DC25, (**d**) CH25_vs_DI25, (**e**) CH25_vs_DC25, and (**f**) DI25_vs_DC25.

Compared to the control group, exposure to chlorothalonil (Ctr_vs_CH10 and Ctr_vs_CH25) mainly caused immunodeficiency disease (such as autoimmune thyroid disease and systemic lupus erythematosus) caused by endocrine disorders (endocrine resistance, and parathyroid hormone synthesis, secretion, and action) and signaling pathway disorders (estrogen signaling pathway and TGF-beta signaling pathway) (Figure 7 and Figure 8 and Tables S25 and S26). The endocrine system is considered vitally important in frailty because it has a direct or indirect relationship with the immune system [39]. For example, the secretion of estrogen has a positive effect on women’s health and longevity [40]. In the present study, all genes related to the KEGG of the estrogen signaling pathway (*GNAI1*, *HSPA6*, *HSPA1A*, and *HSPA1B*) and longevity regulating pathway-multiple species (*HSPA6, HSPA1A,* and *HSPA1B*) were upregulated when exposed to chlorothalonil. Heat shock protein family A (*ASPA*) proteins play important roles in regulating cell functions during carcinogenesis. Previous studies indicated that the upregulation of *HSPA6*, *HSPA1A*, and *HSPA1B* not only promoted the risk of tumorigenesis and nontumor-related diseases but also increased the probability of poor prognosis [41,42]. In addition, exposure to chlorothalonil affected the estrogen signaling pathway, so it is necessary to further study whether chlorothalonil poses a greater risk to females than to males.

As shown in Figure 7 and Figure 8 and Tables S27 and S28, the KEGG enrichment analysis indicated that exposure to difenoconazole (Ctr_vs_DI10 and Ctr_vs_DI25) could also cause immunodeficiency diseases, such as systemic lupus erythematosus and primary immunodeficiency. There were 6 DEGs enriched in systemic lupus erythematosus when the exposure dose was EC_10_, and the number of DEGs increased to 18 when the exposure dose was EC_25_. These DEGs mainly refer to histone genes such as *H4C1/2/5/8/11/13*, *H3C8/12/13*, *H2BC7/17*, and *H2AC7/14*. Notably, there were 4 DEGs enriched in complement and coagulation cascades when the exposure dose was EC_10_, and the DEGs increased to 10 when the exposure dose was EC_25_. In particular, two important genes, *VWF* and *SERPINE1*, were downregulated in both comparisons of Ctr_vs_DI10 and Ctr_vs_DI25. VWF is a plasma glycoprotein that is crucial for normal platelet thrombosis during hemostasis [43]. *SERPINE1* has been proposed as the key indicator for carcinogenesis and poor prognosis, and the downregulation of *SERPINE1* may be related to the decrease in neutrophils and macrophages in the human body [44]. In addition, the KEGG pathway analysis indicated that exposure to chlorothalonil or difenoconazole could cause the differential expression of genes related to diabetes. Exposure to chlorothalonil mainly refers to type I diabetes mellitus, but exposure to difenoconazole mainly refers to type II diabetes mellitus, which indicates that there are differences in toxicity mechanisms between these two pesticides.

As shown in Figure 7 and Figure 8 and Tables S29 and S30, the most significant (*p* value ≤ 0.05) top three KEGG pathways for the Ctr_vs_DC10 comparison were transcriptional misregulation in cancer, fatty acid degradation, and riboflavin metabolism, but for the Ctr_vs_DC25 comparison, the most significant top three KEGG pathways were cell adhesion molecules, ECM-receptor interaction, and viral protein interaction with cytokine and cytokine receptor. Although some DEGs were enriched in the same KEGG pathway at different exposure doses (EC_10_ and EC_25_), the DEGs were different. For example, the genes enriched in transcriptional misregulation in cancer, *PBX1*, *H3C10*, and *H3C2*, were significantly differentially expressed in the comparison of Ctr_vs_DC10 but not in the comparison of Ctr_vs_DC25. In addition, the genes *TNFRSF13B*, *CD86*, and *IL2RB* were significantly differentially expressed in the comparison of Ctr_vs_DC25 but not in the comparison of Ctr_vs_DC10. Similar to the cell activity results, the results indicated that joint exposure to low and high doses of chlorothalonil and difenoconazole may have different toxic effects on cells.

### 3.6. Quantitative Real-Time PCR Validation

**Figure 9:**
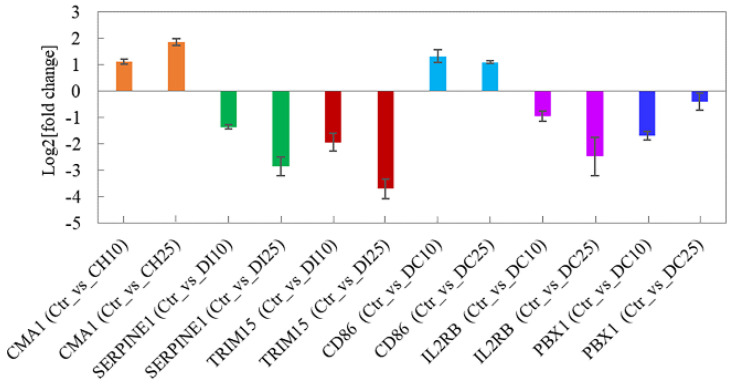
Quantitative real-time PCR results for the selected genes. Data are expressed as the mean ± SD from two independent experiments.

To further validate the transcriptomics results, six differentially expressed genes, *CMA1*, *SERPINE1*, *TRIM15*, *CD86*, *IL2RB*, and *PBX1*, associated with immune regulation [35,36,44,45,46,47] were selected for quantitative real-time PCR validation. As shown in Figure 9, although there were certain differences in fold changes, the differential expression trends of the six genes were consistent with the results of the transcriptomics analysis, which indicated that the transcriptomics results were reliable.

## 4. Conclusions

In this study, the immunotoxicity of 10 commonly detected pesticides in fruits and vegetables was evaluated by using the human Jurkat T-cell line. Difenoconazole and chlorothalonil showed obvious inhibitory effects on cell growth, and the EC_50_ values were 24.66 and 1.17 mg/L, respectively. The joint exposure to these two pesticides showed a synergistic effect at low concentrations (total dose lower than 10.58 mg/L) but an antagonistic effect at high concentrations (total dose higher than 10.58 mg/L). The transcriptomics analysis indicated that the DEGs for a single exposure to chlorothalonil mainly affected cation transportation, extracellular matrix organization, and leukocyte cell adhesion. However, exposure to difenoconazole mainly affected nervous system development, muscle contraction, and immune system processes. However, when the joint exposure dose was EC_10_, the DEGs were mainly involved in the formation of cell structures, but when exposed at EC_25_, the DEGs were mainly involved in cellular processes and metabolism. Although Jurkat T-cell line models were successfully used to explore the immunotoxicity of different pesticides, and the single and combined immunotoxicological mechanisms of difenoconazole and chlorothalonil were studied, this study did not conduct animal experiments, so the toxicity results cannot be extrapolated to humans. Therefore, further animal experiments are needed to verify the results.

## Data Availability

The data presented in this article are available on reasonable request from the corresponding author.

## Supplementary Materials

The following supporting information can be downloaded at: https://www.mdpi.com/article/10.3390/foods13010034/s1.
